# Supplementary material for: Human antibodies against West Nile and related orthoflaviviruses
Source: bioRxiv. 2026 Apr 6:2026.04.02.715800. Preprint. [Version 1] doi: 10.64898/2026.04.02.715800 (PMC13081833; doi:10.64898/2026.04.02.715800)
Supplement: Supplement 1 — Figure S1. Demographics and serologic features of individuals hospitalized for WND/WNF. Related to main Figure 1. (A) Demographic characteristics of the cohort. (B) Geographic distribution of the patients’ residency. Case distribution shows the involvement of 11 Districts, with clusters in Central Serbia in 2022 and Northern Serbia in 2023. Blue dots indicate 2022 cases (with a dominant cluster near the city of Kragujevac, Šumadija District), while red dots represent the residency of the 2023 cases (with a dominant cluster near the city of Novi Sad). The map was generated with QGIS v3.12 (QGIS Development Team, 2020) starting from the GADM database (v4.1, July 2022, https://gadm.org/). (C) Comparison between NanoLuc and Renilla reporter virus particles (RVPs). WNVRVP were produced side-by-side by transfecting packaging cells with a NanoLuc- or Renilla-expressing WNV replicon (see Materials and Methods). Different volumes of supernatant-containing RVPs harvested at 24h, 48h and 72h were added to Huh 7.5 cells and the luminescence measured from cell lysates. The lower background with NanoLuc results in improved signal-to-noise ratio. Dotted horizontal lines indicate the average luminescence that was measured in the absence of WNVRVP. (D) Correlation between serum IgG binding to WNVEDIII and NT50 values. AUC is Area Under the Curve of ELISA binding. Pearson correlation. (E) Correlation between serum IgG binding to WNVEDIII and age at hospitalization. Pearson correlation. (F) Correlation between serum IgG binding to WNVEDIII and time from symptoms onset to serum sampling. Pearson correlation. (G) Comparison of serum IgG binding to WNVEDIII between samples from male and female participants. Unpaired two-tailed t-test. (H) Correlation between serum IgG binding to WNVEDIII and days of hospitalization. Pearson correlation. (I) Comparison of serum IgG binding to WNVEDIII between samples from survivors and non-survivors. Unpaired two-tailed t-test. (J) Correlation between WNV [file media-1.pdf]

Figure S1

A

Cohort	Age	Gender			
		Male		Female	
		n	%	n	%
**WND**	59.10 years (CI 95%: 55.145 – 63.055)	45	65.21	24	34.78
**WNF**	42.33 years (CI 95%: 31.99 - 52.68)	1	33.33	2	66.66

B

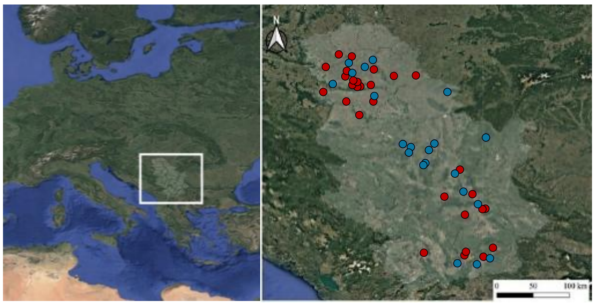

C

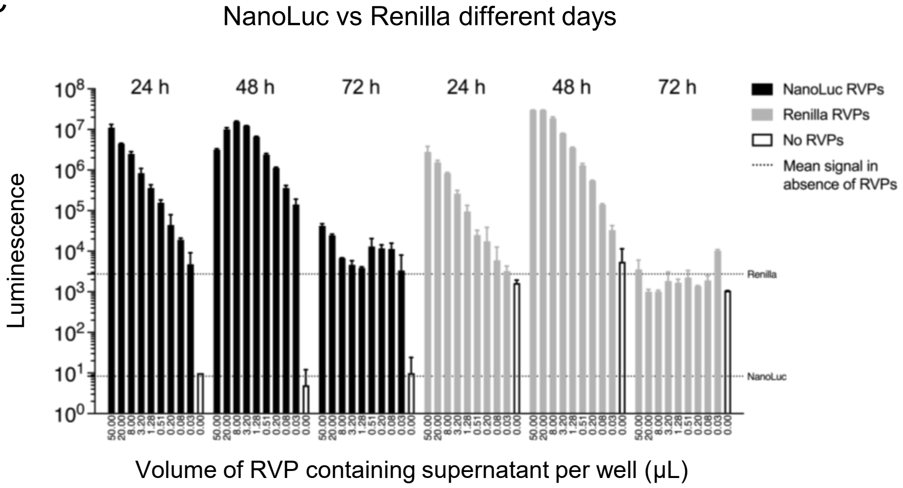

D

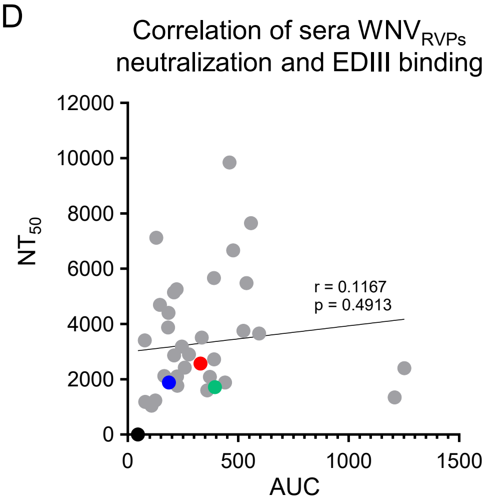

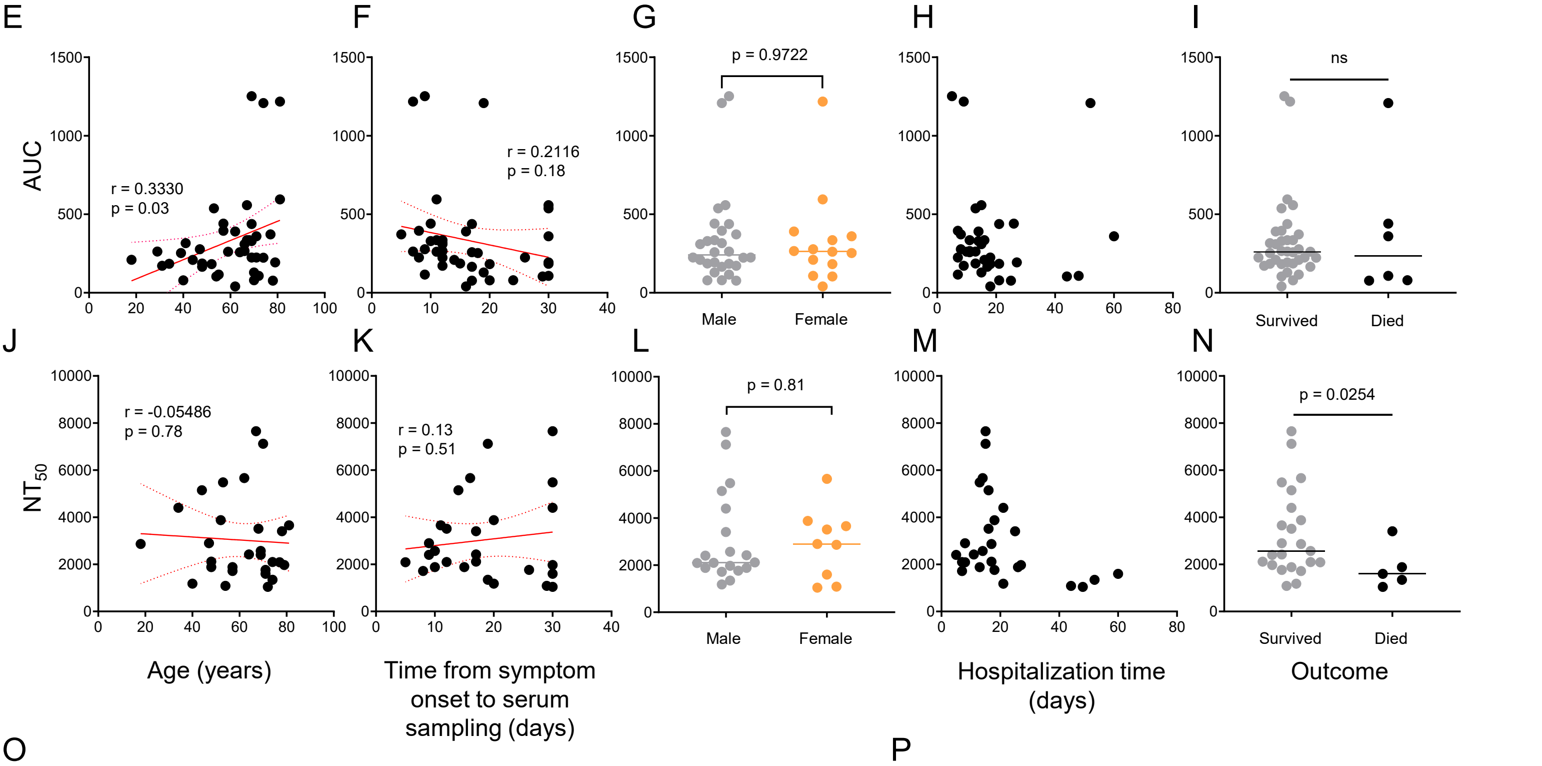

O

anti-IFN-α2 autoantibodies					anti-IFN-ω autoantibodies				
Age group	Serbian cohort	General population	p-value	Significantly higher?	Age group	Serbian cohort	General population	p-value	Significantly higher?
<50	0 / 8 (0%)	8 / 2710 (0.30%)	—	No	<50	0 / 8 (0%)	40 / 4446 (0.90%)	—	No
50–60	2 / 7 (28.6%)	3 / 1736 (0.17%)	< 0.001	Yes	50–60	1 / 7 (14.3%)	13 / 1736 (0.75%)	0.004	Yes
60–70	4 / 12 (33.3%)	14 / 2475 (0.57%)	< 0.001	Yes	60–70	4 / 12 (33.3%)	12 / 2475 (0.48%)	< 0.001	Yes
70–80	1 / 10 (10.0%)	29 / 1790 (1.62%)	0.15	No	70–80	1 / 10 (10.0%)	29 / 1790 (1.62%)	0.15	No
80–90	0 / 2 (0%)	79 / 1580 (5.00%)	—	No	80–90	0 / 2 (0%)	56 / 1580 (3.54%)	—	No
**50–80**	**7 / 29 (24.1%)**	**46 / 6001 (0.766%)**	**< 0.001**	**Yes**	**50–80**	**6 / 29 (20.6%)**	**54 / 6001 (0.899%)**	**< 0.001**	**Yes**
P-values from two-sample, one-sided Fisher's exact tests									
"Significantly higher" defined as p < 0.05									
"—" indicates no test due to zero events in the cohort									

P

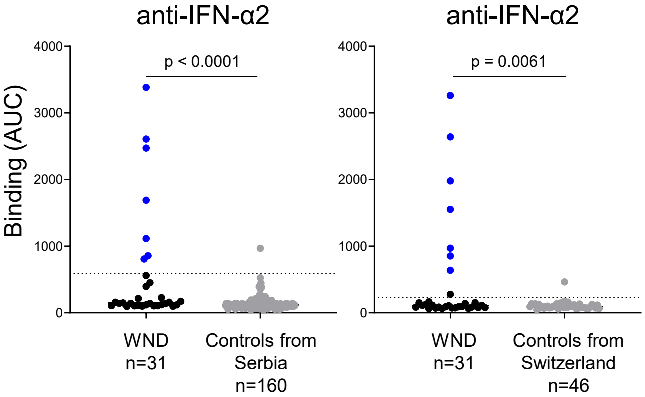

Figure S2

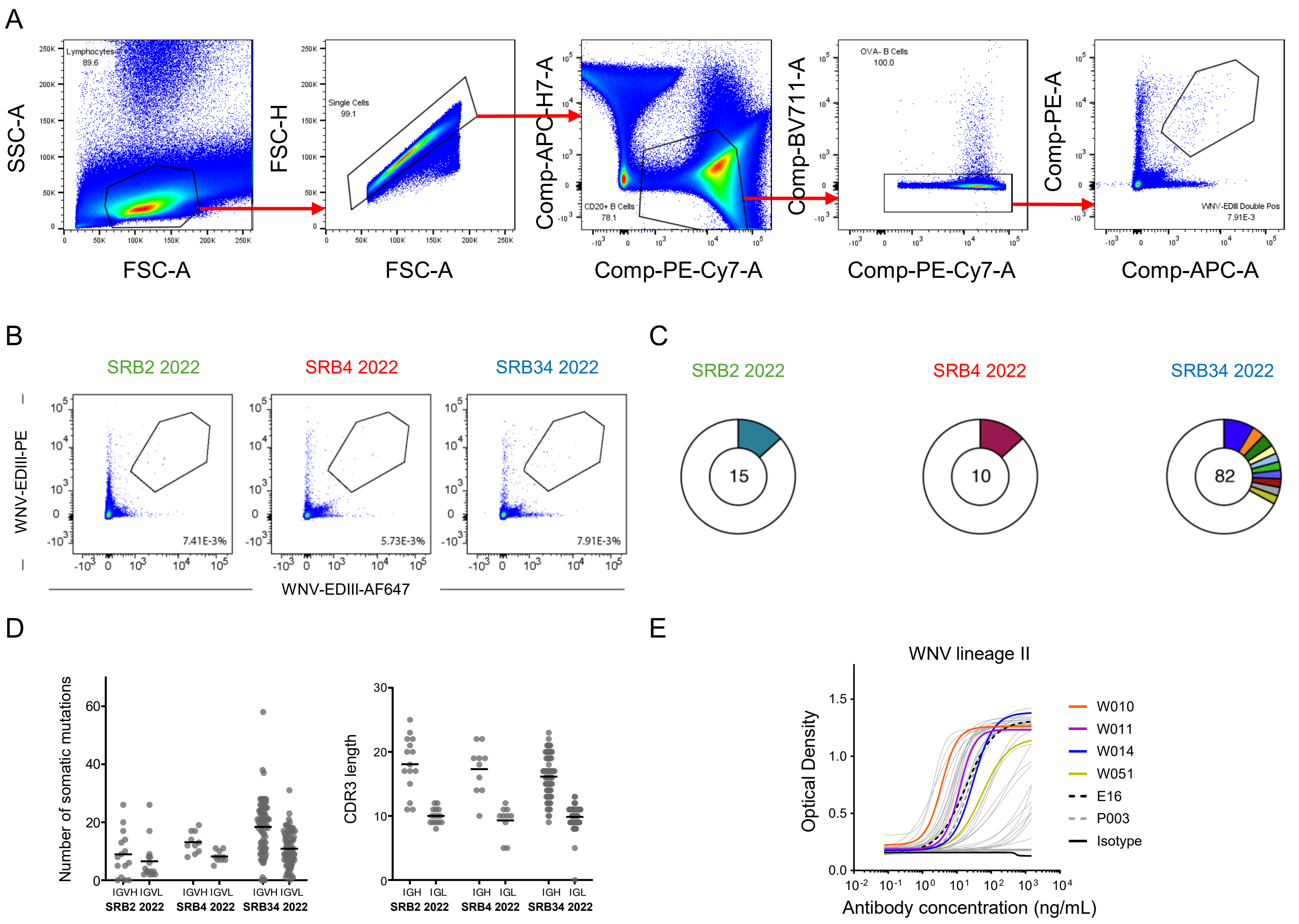
A
Lymphocytes 89.6
SSC-A
FSC-A
Single Cells 99.1
FSC-H
FSC-A
CD20+ B Cells 78.1
Comp-APC-H7-A
Comp-PE-Cy7-A
OVA- B Cells 100.0
Comp-BV711-A
Comp-PE-Cy7-A
WNV-EDIII Double Pos 7.91E-3
Comp-PE-A
Comp-APC-A
B
SRB2 2022
SRB4 2022
SRB34 2022
7.41E-3%
5.73E-3%
7.91E-3%
WNV-EDIII-PE
WNV-EDIII-AF647
C
SRB2 2022
SRB4 2022
SRB34 2022
15
10
82
D
Number of somatic mutations
IGVH IGVL
SRB2 2022
SRB4 2022
SRB34 2022
CDR3 length
IGH IGL
E
WNV lineage II
Optical Density
Antibody concentration (ng/mL)
W010
W011
W014
W051
E16
P003
Isotype

Figure S3

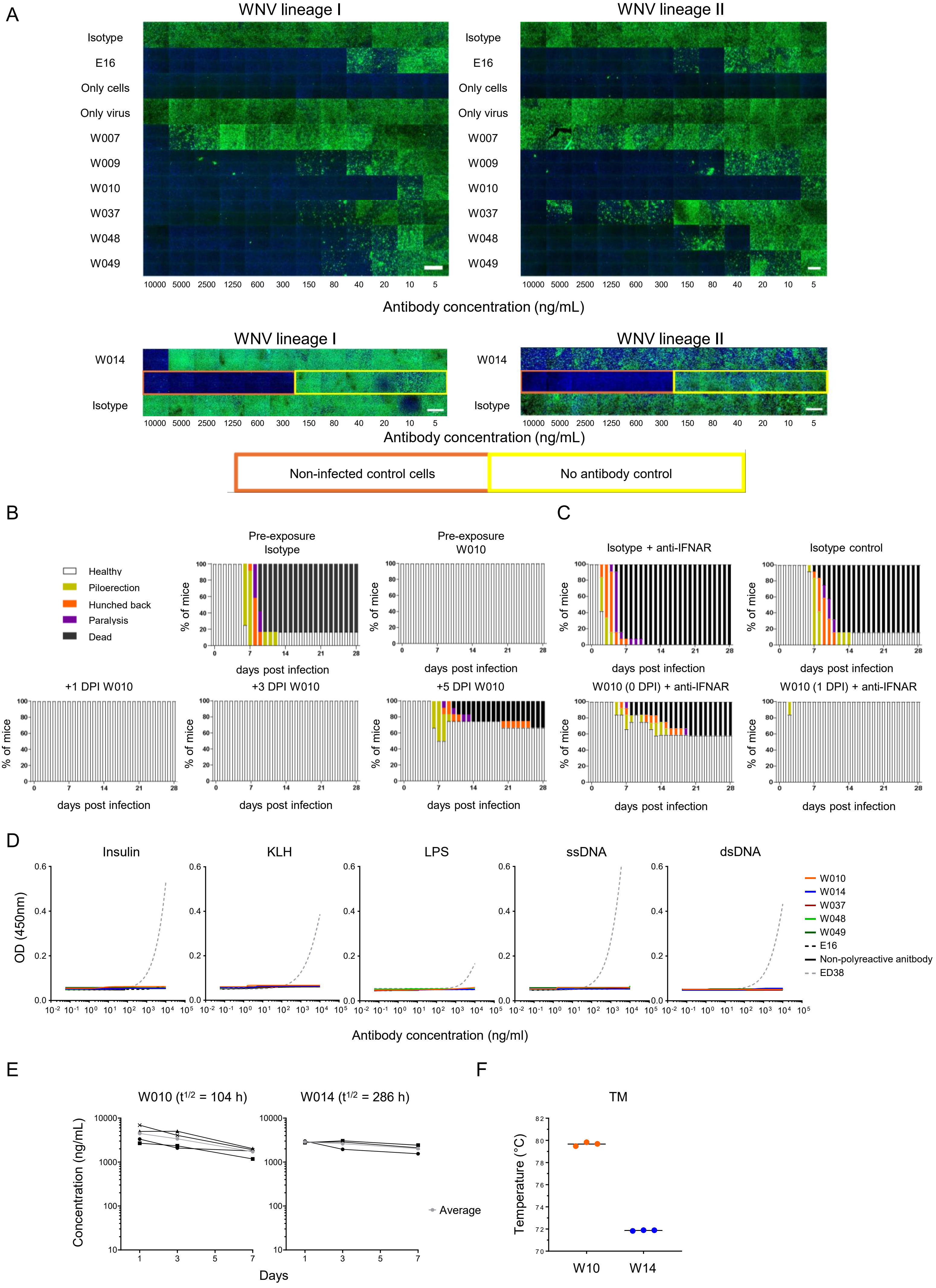

A
WNV lineage I
WNV lineage II
Isotype
E16
Only cells
Only virus
W007
W009
W010
W037
W048
W049
10000 5000 2500 1250 600 300 150 80 40 20 10 5
Antibody concentration (ng/mL)
W014
Isotype
Non-infected control cells
No antibody control
B
C
Healthy
Piloerection
Hunched back
Paralysis
Dead
Pre-exposure Isotype
Pre-exposure W010
Isotype + anti-IFNAR
Isotype control
+1 DPI W010
+3 DPI W010
+5 DPI W010
W010 (0 DPI) + anti-IFNAR
W010 (1 DPI) + anti-IFNAR
% of mice
days post infection
D
Insulin
KLH
LPS
ssDNA
dsDNA
OD (450nm)
Antibody concentration (ng/ml)
W010
W014
W037
W048
W049
E16
Non-polyreactive anitbody
ED38
E
W010 (t1/2 = 104 h)
W014 (t1/2 = 286 h)
Concentration (ng/mL)
Days
Average
F
TM
Temperature (°C)
W10
W14

Figure S4

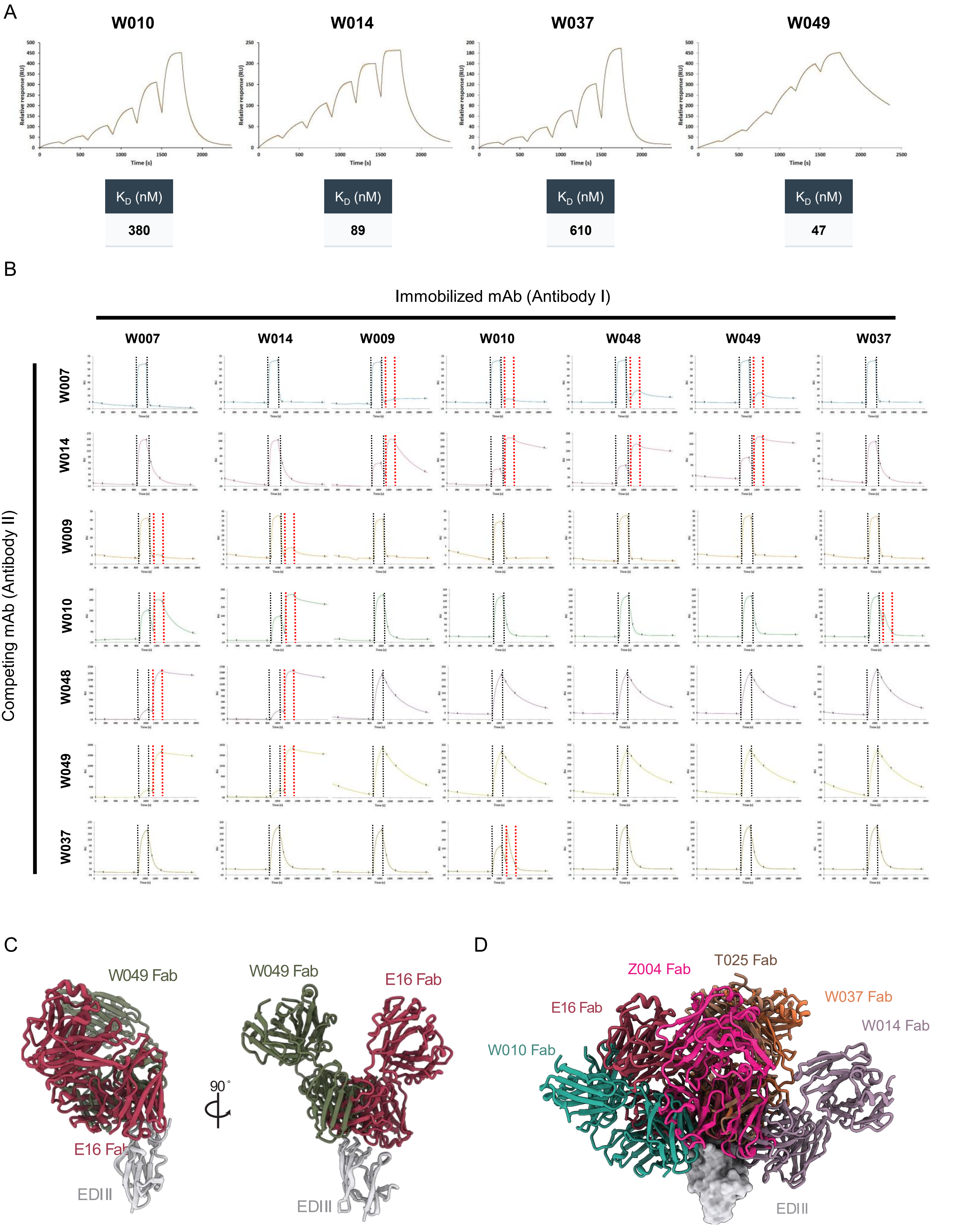
A
W010
W014
W037
W049
Relative response (RU)
Time (s)
K_D (nM)
380
K_D (nM)
89
K_D (nM)
610
K_D (nM)
47
B
Immobilized mAb (Antibody I)
W007
W014
W009
W010
W048
W049
W037
Competing mAb (Antibody II)
W007
W014
W009
W010
W048
W049
W037
C
W049 Fab
E16 Fab
EDIII
90˚
W049 Fab
E16 Fab
EDIII
D
Z004 Fab
T025 Fab
W037 Fab
E16 Fab
W014 Fab
W010 Fab
EDIII

Figure S5

A

B

C

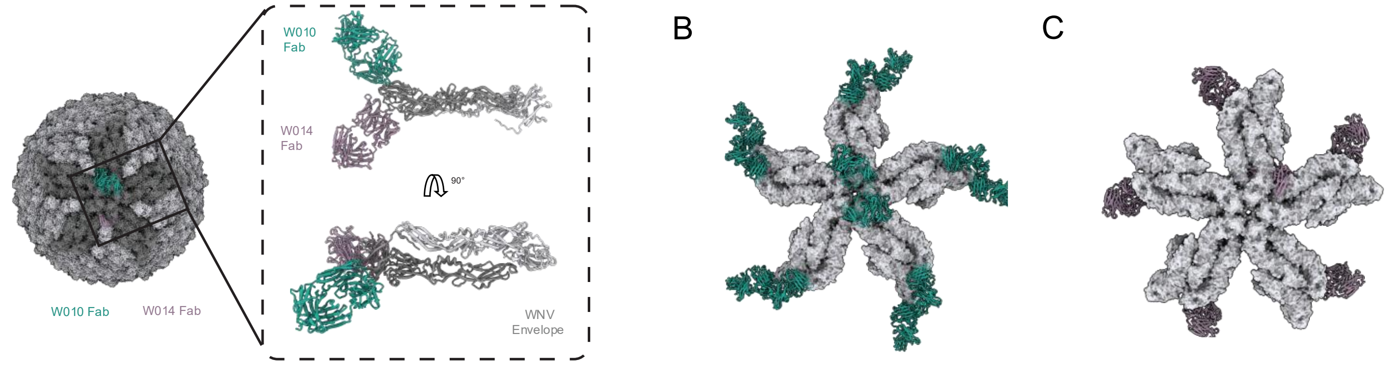

D

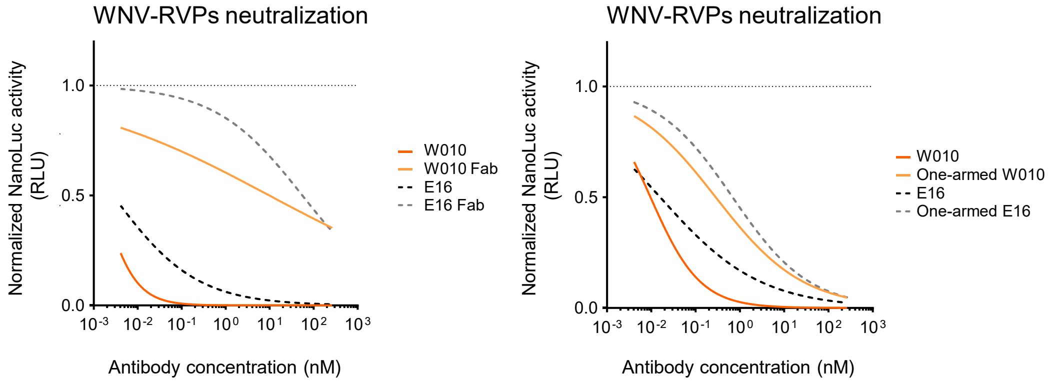

Molecule	IC_{50} value (nM)	MNR
W010 mAb	0.00147	6673.5
W010 Fab	9.81	
E16 mAb	0.00273	20315.01
E16 Fab	55.46	

Molecule	IC_{50} value (nM)	MNR
W010 mAb	0.009587	29.66
One-armed W010	0.2844	
E16 mAb	0.01586	43.12
One-armed E16	0.6840	

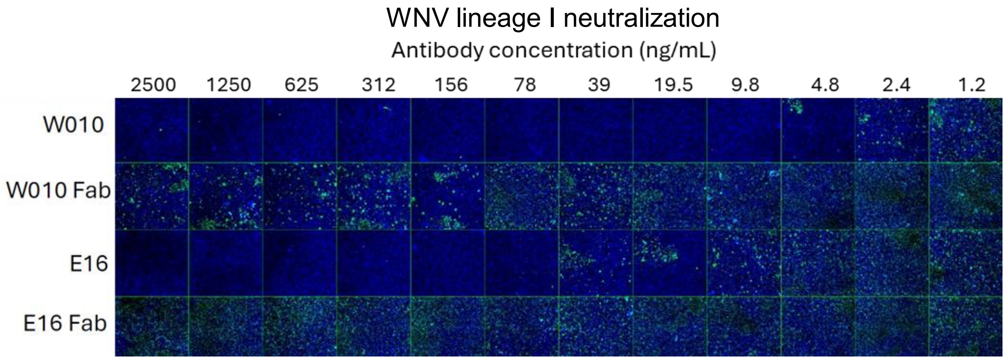

E

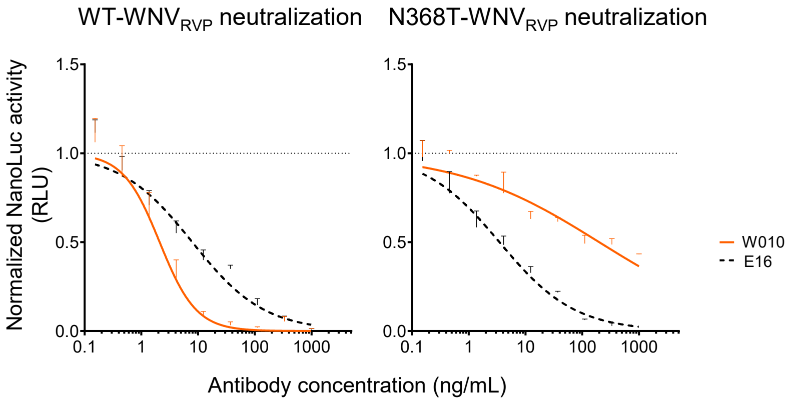

Figure S6

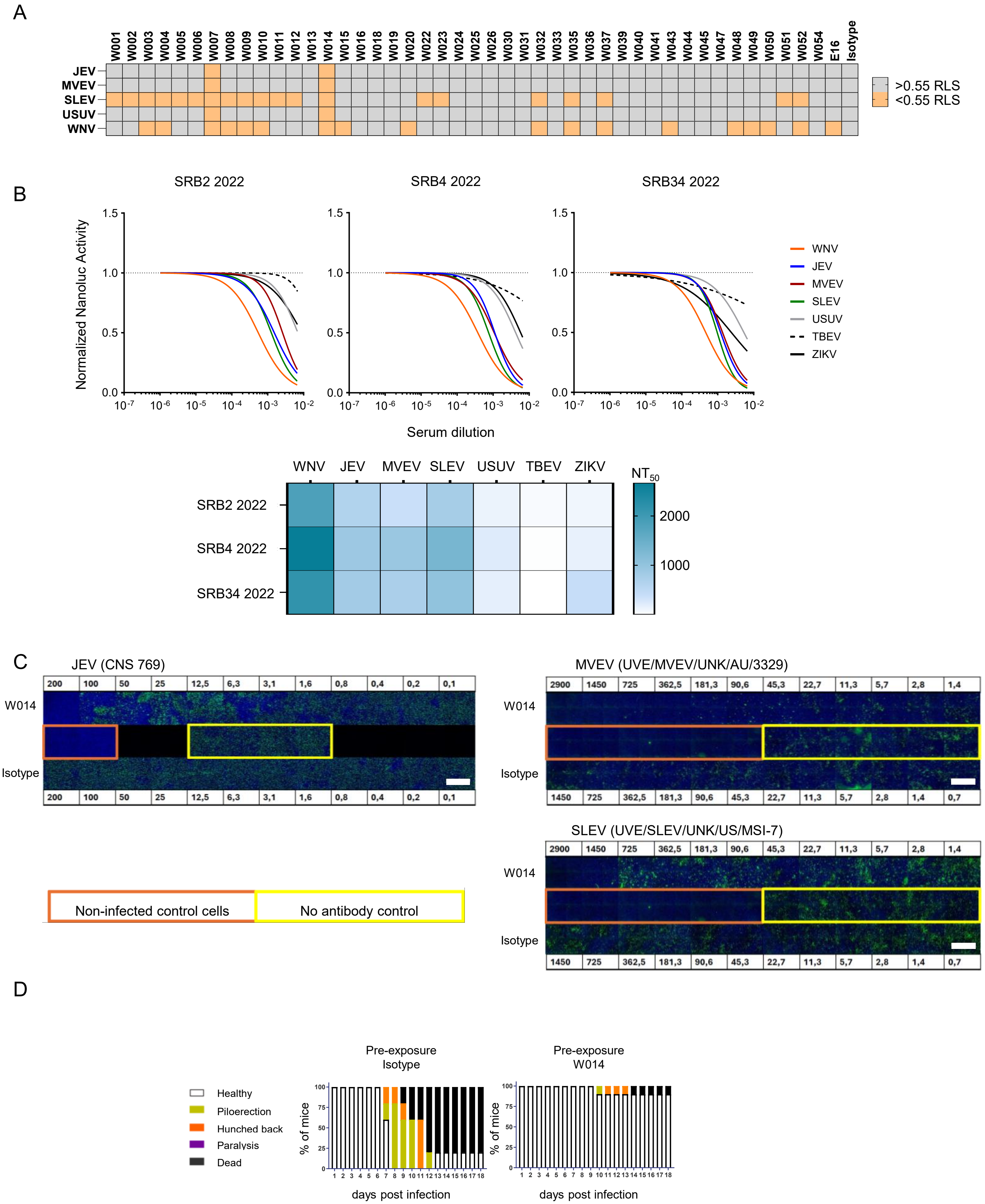
A
W001 W002 W003 W004 W005 W006 W007 W008 W009 W010 W011 W012 W013 W014 W015 W016 W018 W019 W020 W022 W023 W024 W025 W026 W030 W031 W032 W033 W035 W036 W037 W039 W040 W041 W043 W044 W045 W047 W048 W049 W050 W051 W052 W054 E16 Isotype
JEV
MVEV
SLEV
USUV
WNV
>0.55 RLS
<0.55 RLS
B
SRB2 2022
SRB4 2022
SRB34 2022
Normalized Nanoluc Activity
Serum dilution
WNV
JEV
MVEV
SLEV
USUV
TBEV
ZIKV
NT50
2000
1000
C
JEV (CNS 769)
MVEV (UVE/MVEV/UNK/AU/3329)
SLEV (UVE/SLEV/UNK/US/MSI-7)
W014
Isotype
Non-infected control cells
No antibody control
D
Pre-exposure Isotype
Pre-exposure W014
Healthy
Piloerection
Hunched back
Paralysis
Dead
% of mice
days post infection
