# Supplementary material for: Human antibodies against West Nile and related orthoflaviviruses
Source: bioRxiv. 2026 Apr 6:2026.04.02.715800. Preprint. [Version 1] doi: 10.64898/2026.04.02.715800 (PMC13081833; doi:10.64898/2026.04.02.715800)
Supplement: Supplement 2 — Table S1. Characteristics of study participants. [file media-2.pdf]

Table S1. Characteristics of study participants (n=72).

Participant ID	Gender (M/F)	Age at diagnosis (years)	Residency (place)	Suspected disease (WNF/WND)	Clinical diagnosis	High serum neutralization in the screening (yes/no)	Anti-type I IFN neutralization (IFNα2, IFNω, both)
SRB1 2022	M	57	Kragujevac	WND	Viral meningoencephalitis (WNV)	yes	both
SRB2 2022	M	57	Kragujevac	WND	Viral meningitis (WNV)	yes	n.d.
SRB3 2022	M	77	Aranđelovac	WND	Viral meningoencephalitis (WNV)	yes	n.d.
SRB4 2022	M	69	Topola	WND	Viral meningoencephalitis (WNV)	yes	n.d.
SRB5 2022	F	66	Velika Plana	WND	Viral meningoencephalitis (WNV)	no	n.d.
SRB6 2022	F	68	Aranđelovac	WND	Viral meningoencephalitis (WNV)	yes	n.d.
SRB7 2022	M	67	Topola	WND	Viral meningoencephalitis (WNV)	no	n.d.
SRB8 2022	M	66	Kragujevac	WND	Viral meningoencephalitis (WNV)	no	n.d.
SRB9 2022	F	59	Rača	WND	Viral meningoencephalitis (WNV)	no	n.d.
SRB10 2022	F	81	Kragujevac	WND	Viral meningoencephalitis (WNV)	no	n.d.
SRB11 2022	M	31	Topola	WND	Viral meningoencephalitis (WNV)	no	n.d.
SRB12 2022	M	69	Paraćin	WND	Viral meningoencephalitis (WNV)	no	n.d.
SRB14 2022	M	50	Kragujevac	WND	Viral meningoencephalitis	yes	NT
SRB15 2022	M	64	Kragujevac	WND	Viral meningoencephalitis (WNV)	yes	both
SRB16 2022	F	47	Kragujevac	WNF	West Nile fever	yes	NT
SRB17 2022	M	29	Aranđelovac	WND	Viral meningoencephalitis (WNV)	no	n.d.
SRB18 2022	M	55	Topola	WND	Viral meningoencephalitis (WNV)	no	n.d.
SRB21 2022	M	64	Donji Ljubeš	WND	Status febrilis, encephalopathia	yes	NT
SRB22 2022	M	49	Vrtište	WND	Viral encephalitis	no	NT
SRB23 2022	F	39	Niš	WNF	West Nile fever	no	NT
SRB24 2022	M	41	Niš	WNF	West Nile fever	no	NT
SRB25 2022	M	78	Gnjilane	WND	Viral encephalitis	no	NT
SRB26 2022	F	63	Majdanpek	WND	Viral encephalitis	no	NT
SRB27 2022	M	73	Vranjska banja	WND	Viral encephalitis	no	NT
SRB28 2022	M	66	Bujanovac	WND	Viral encephalitis	no	NT
SRB29 2022	M	68	Bačka Palanka	WND	Viral encephalitis	no	NT
SRB30 2022	F	76	Bečej	WND	Viral encephalitis	no	NT
SRB31 2022	F	54	Nadalj	WND	Viral encephalitis (WNV)	yes	n.d.
SRB32 2022	M	63	Stepanovićevo	WND	Viral encephalitis	no	NT
SRB33 2022	M	48	Novi Sad	WND	Viral encephalitis (WNV)	yes	n.d.
SRB34 2022	M	48	Inđija	WND	Viral encephalitis (WNV)	yes	n.d.
SRB35 2022	F	62	Novi Sad	WND	ncephalitis (WNV) and non-Hodgkin lyn	no	n.d.
SRB36 2022	M	40	Vrbas	WND	Viral encephalitis (WNV)	yes	n.d.
SRB37 2022	M	29	Novi Sad	WND	Viral encephalitis	no	NT
SRB38 2022	F	72	Golubinci	WND	Viral encephalitis (WNV)	yes	n.d.
SRB1 2023	M	53	Grabovci	WND	WNV encephalitis	yes	IFNa2
SRB2 2023	F	62	Sečanj	WND	WNV encephalitis	yes	n.d.
SRB3 2023	F	52	Šid	WND	WNV encephalitis	yes	n.d.
SRB4 2023	M	70	Odžaci	WND	WNV encephalitis	no	n.d.
SRB5 2023	M	44	Čurug	WND	WNV encephalitis	yes	n.d.
SRB6 2023	F	81	Sremska Kamenica	WND	WNV encephalitis	yes	n.d.
SRB7 2023	M	67	Ravno Selo	WND	WNV encephalitis	yes	both
SRB8 2023	M	88	Futog	WND	Encephalitis viralis non-specificata	yes	NT
SRB9 2023	M	79	Ledinci	WND	WNV encephalitis	yes	n.d.
SRB10 2023	M	74	Golubinci	WND	WNV encephalitis	yes	n.d.
SRB11 2023	M	71	Novi Sad	WND	WNV encephalitis	yes	n.d.
SRB12 2023	M	70	Novi Sad	WND	WNV encephalitis	yes	n.d.
SRB13 2023	M	34	Feketić	WND	Meningoencephalitis WNV	yes	n.d.
SRB14 2023	M	69	Prokuplje	WND	WNV encephalitis	yes	both
SRB15 2023	F	18	Nova Crvenka	WND	Meningoencephalitis WNV	yes	n.d.
SRB16 2023	M	78	Kovilj	WND	Encephalitis acuta	yes	NT
SRB17 2023	M	64	Zrenjanin	WND	Encephalitis viralis non-specificata	yes	NT
SRB18 2023	M	62	Rumenka	WND	Encephalitis viralis non-specificata	yes	NT
SRB19 2023	M	72	Bački Petrovac	WND	Encephalomyelitis	yes	NT
SRB20 2023	M	78	Šid	WND	WNV encephalitis	yes	n.d.
SRB21 2023	M	74	Futog	WND	WNV encephalitis	yes	both
SRB22 2023	M	69	Sremska Mitrovica	WND	WNV encephalitis	no	both
SRB23 2023	F	71	Sremska Kamenica	WND	WNV encephalitis	yes	n.d.
SRB24 2023	F	35	Niš	WND	Encephalitis viralis non-specificata	no	NT
SRB25 2023	F	75	Niš	WND	Encephalitis viralis non-specificata	no	NT
SRB26 2023	F	55	Vranje	WND	Encephalitis viralis non-specificata	no	NT
SRB27 2023	F	49	Niš	WND	Encephalitis viralis non-specificata	no	NT
SRB28 2023	M	24	Kostadince	WND	Encephalitis viralis non-specificata	no	NT
SRB29 2023	F	64	Sastavar	WND	Encephalitis viralis non-specificata	no	NT
SRB30 2023	M	77	Niš	WND	Meningoencephalitis acuta	no	NT
SRB31 2023	M	30	Glogovac	WND	Encephalitis viralis non-specificata	no	NT
SRB32 2023	F	59	Niš	WND	Encephalopathia	no	NT
SRB33 2023	F	28	Niš	WND	Encephalitis viralis non-specificata	no	NT
SRB34 2023	F	28	Aleksinac	WND	Encephalitis viralis non-specificata	yes	NT
SRB35 2023	M	18	Vladičin Han	WND	Encephalitis viralis non-specificata	no	NT
SRB36 2023	F	61	Doljane	WND	Encephalitis viralis non-specificata	no	NT
SRB37 2023	F	57	Popovac	WND	Encephalitis viralis non-specificata	no	NT

NT = Not Tested
n.d. = non detectable
ND = no data

Time from symptom onset to serum sampling (days)	Duration of hospitalization (days)	Outcome (survived/died)	Clinical Center
10	26	Died	Clinical Center Kragujevac
8	7	Survived	Clinical Center Kragujevac
5	8	Survived	Clinical Center Kragujevac
10	14	Survived	Clinical Center Kragujevac
12	11	Survived	Clinical Center Kragujevac
12	16	Survived	Clinical Center Kragujevac
11	11	Survived	Clinical Center Kragujevac
12	15	Survived	Clinical Center Kragujevac
11	10	Survived	Clinical Center Kragujevac
7	9	Survived	Clinical Center Kragujevac
12	9	Survived	Clinical Center Kragujevac
8	7	Survived	Clinical Center Kragujevac
9	15	Survived	Clinical Center Kragujevac
17	11	Survived	Clinical Center Kragujevac
9	8	Survived	Clinical Center Kragujevac
7	13	Survived	Clinical Center Kragujevac
9	7	Survived	Clinical Center Kragujevac
12	12	Survived	Clinical Center Niš
11	ND	ND	Clinical Center Niš
18	ND	Survived	Clinical Center Niš
19	ND	Survived	Clinical Center Niš
5	ND	ND	Clinical Center Niš
4	ND	ND	Clinical Center Niš
6	ND	ND	Clinical Center Niš
5	ND	ND	Clinical Center Niš
4	10	Survived	Clinical Center Novi Sad
9	ND	ND	Clinical Center Novi Sad
29	44	Survived	Clinical Center Novi Sad
7	ND	ND	Clinical Center Novi Sad
17	17	Survived	Clinical Center Novi Sad
15	13	Survived	Clinical Center Novi Sad
16	18	Survived	Clinical Center Novi Sad
20	21	Survived	Clinical Center Novi Sad
17	ND	ND	Clinical Center Novi Sad
30	48	Died	Clinical Center Novi Sad
30	13	Survived	Clinical Center Novi Sad
16	14	Survived	Clinical Center Novi Sad
20	18	Survived	Clinical Center Novi Sad
24	21	Died	Clinical Center Novi Sad
14	16	Survived	Clinical Center Novi Sad
11	ND	Survived	Clinical Center Novi Sad
30	15	Survived	Clinical Center Novi Sad
ND	14	Survived	Clinical Center Novi Sad
30	27	Survived	Clinical Center Novi Sad
12	7	Survived	Clinical Center Novi Sad
26	18	Survived	Clinical Center Novi Sad
19	15	Survived	Clinical Center Novi Sad
30	21	Survived	Clinical Center Novi Sad
9	5	Survived	Clinical Center Novi Sad
ND	17	Survived	Clinical Center Novi Sad
15	17	Survived	Clinical Center Novi Sad
20	20	Survived	Clinical Center Novi Sad
25	28	Survived	Clinical Center Novi Sad
16	16	Survived	Clinical Center Novi Sad
17	25	Died	Clinical Center Novi Sad
19	52	Died	Clinical Center Novi Sad
17	21	Survived	Clinical Center Novi Sad
30	60	Died	Clinical Center Novi Sad
5	ND	ND	Clinical Center Niš
5	ND	ND	Clinical Center Niš
5	ND	ND	Clinical Center Niš
8	ND	ND	Clinical Center Niš
4	ND	ND	Clinical Center Niš
3	ND	ND	Clinical Center Niš
5	ND	ND	Clinical Center Niš
5	ND	ND	Clinical Center Niš
2	ND	ND	Clinical Center Niš
5	ND	ND	Clinical Center Niš
8	20	Survived	Clinical Center Niš
10	ND	ND	Clinical Center Niš
7	ND	ND	Clinical Center Niš
10	ND	ND	Clinical Center Niš
