# Supplementary material for: Human antibodies against West Nile and related orthoflaviviruses
Source: bioRxiv. 2026 Apr 6:2026.04.02.715800. Preprint. [Version 1] doi: 10.64898/2026.04.02.715800 (PMC13081833; doi:10.64898/2026.04.02.715800)
Supplement: Supplement 3 — Table S2. Effective and inhibitory concentrations of recombinantly expressed monoclonal antibodies. [file media-3.pdf]

Table S3. Effective and inhibitory concentrations of recombinantly expressed monoclonal antibodies

	EC$_{50}$ (ng/mL)					
	WNV lin I - EDIII	WNV lin II - EDIII	JEV - EDIII	MVEV - EDIII	SLEV - EDIII	USUV - EDIII
W001						
W002						
W003						
W004	132	83.6				
W005	2.325	2.092				
W006						
W007						
W008	20.6	10.66				
W009	4.281	2.204				
W010	4.312	3.532				
W011	9.398	11.88	17.27	203.1		17.89
W012	6.792	3.747				
W013						
W014	42.22	30.06	31.41		15.94	17.84
W015	14.42	7.982				
W016	9.385	8.695				
W018	13.04	2.149				
W019	64.5	27.95				
W020						
W022	9.456	6.684				
W023	13.79	10.75				
W024						
W025	89.45	22.09				
W026						
W030						
W031						
W032						
W033						
W035	33.42	16.65				
W036						
W037	17.54	19.38				
W039						
W040	173.6	129				
W041						
W043	8.256	7.048				
W044	23.33	11.12				
W045	18.46	8.676				
W047	4.958	7.519				
W048	4.603	6.017				
W049	14.54	17.2				
W050	97.35	86.75				
W051	53.47	53.68			17.19	
W052						
W054	13.18	13.82				

Not-determined
Not-tested
Non-neutralizing

IC$_{50}$ (ng/mL) - RVPs					IC$_{50}$ (ng/mL) - virus	
WNV$_{RVPs}$ lin I	JEV$_{RVPs}$	MVEV$_{RVPs}$	SLEV$_{RVPs}$	USUV$_{RVPs}$	WNV lin I	WNV lin II
90.21	166.8	88.91	13.44	59.82	107.1	85.15
15						
782.1					24.71	18.23
3.696					4.819	3.839
102.2	43.61	27.77	4.327	36.27	772.9	142.9
13.48						
238.3						
152.8						
113.2						
			140.2			
6.696					56.78	35.9
34.25					14.14	13.09
2.028					10.39	11.65
14.11						
1347		485.7	12.8			
212.1						
