# Supplementary material for: Human antibodies against West Nile and related orthoflaviviruses
Source: bioRxiv. 2026 Apr 6:2026.04.02.715800. Preprint. [Version 1] doi: 10.64898/2026.04.02.715800 (PMC13081833; doi:10.64898/2026.04.02.715800)
Supplement: Supplement 4 — Table S3. Crystallographic data processing and refinement statistics. [file media-4.pdf]

Table S3. Crystallographic data processing and refinement statistics

		W010-WNV EDIII	W014-WNV EDIII	W037-WNV EDIII	W049-WNV EDIII
**PDB ID**		9ZRM	9ZRN	9ZRO	9ZRP
**Data collection**[a]					
Space group		P1	P 1 21 1	P 21 21 2	P 21 21 21
Unit cell (Å)		39.9 56.8 67.8	41.1 122.1 54.1	74.9 158.9 52.4	97.179 112.691 129.888
α, β, γ (°)		66.5 89.4 70.2	90 106.331 90	90 90 90	90 90 90
Wavelength (Å)		0.97946	0.97946	0.97946	0.97946
Resolution (Å)		37.14 - 1.8 (1.864 - 1.8)	39.54 - 2.1 (2.175 - 2.1)	37.76 - 1.4 (1.45 - 1.4)	39.55 - 2.5 (2.56 - 2.5)
Unique Reflections		42869 (4320)	29372 (2825)	122938 (11699)	93009 (6498)
Completeness (%)		90.80 (91.79)	98.35 (93.76)	99.38 (95.62)	97.86 (96.99)
Redundancy		3.9 (3.8)	3.6 (3.1)	6.7 (6.3)	6.9 (7)
$CC_{1/2}$ (%)		99.7 (94.3)	99.1 (51.7)	99.9 (62.1)	99.6 (48.7)
<I/σI>		11.9 (3.8)	5.42 (1.38)	12.29 (2.13)	8.7 (1.2)
Mosaicity (˚)		0.12	0.17	0.09	0.15
R_{merge} (%)		6.6 (30.3)	8.579 (60.53)	2.812 (38.43)	14.7 (95.1)
R_{pim} (%)		3.8 (17.9)	8.579 (60.53)	2.812 (38.43)	9.1(63.5)
Wilson *B*-factor		17.6	28.09	13.75	55.18
**Refinement and Validation**					
Resolution (Å)		37.14 - 1.8	39.54 - 2.1	37.76 - 1.4	39.55 - 2.5
Number of atoms					
	Protein	4016	4096	4016	8365
	Ligand	0	0	0	
	Waters	480	367	788	176
R_{work}/R_{free} (%)		17.7/21.6	19.8/25.1	18.3/21.1	20.5/26.2
R.m.s. deviations					
	Bond lengths (Å)	0.004	0.024	0.013	0.008
	Bond angles (˚)	0.78	1	1.3	0.98
MolProbity score		0.98	1.46	1.06	1.78
Clashscore (all atom)		1.88	3.9	1.76	4.75
Poor rotamers (%)		0.44	1.3	0.88	1.64
Ramachandran plot					
	Favored (%)	97.89	96.6	97.3	94.52
	Allowed (%)	2.1	3.7	2.67	5.38
	Disallowed (%)	0	0	0	0.09
Average *B*-factor (Å)		22.36	32.13	19.9	58.41

[a]Numbers in parentheses correspond to the highest resolution shell
